# Adherence Clubs to Improve Hypertension Management in Nigeria: Clubmeds, a Feasibility Study

**DOI:** 10.5334/gh.1109

**Published:** 2022-03-16

**Authors:** Godsent C. Isiguzo, Karla Santo, Rajmohan Panda, Lilian Mbau, Shiva R. Mishra, Collins N. Ugwu, Salim S. Virani, Augustine N. Odili, Emily R. Atkins

**Affiliations:** 1 Alex Ekwueme Federal University Teaching Hospital Abakaliki, Abakaliki, Nigeria; 2 College of Medicine, Ebonyi State University Abakaliki, Nigeria; 3 Westmead Applied Research Centre, Faculty of Medicine and Health, The University of Sydney, Sydney, Australia; 4 The George Institute for Global Health, University of New South Wales, Sydney, Australia; 5 Public Health Foundation of India, New Delhi, India; 6 Kenya Cardiac Society, Nairobi, Kenya; 7 The University of Queensland, Brisbane, Australia; 8 Nepal Development Society, Bharatpur, Nepal; 9 Michael E. DeBakey Veterans Affairs Medical Center & Baylor College of Medicine, Houston, USA; 10 Circulatory Health Research Laboratory, College of Health Sciences, University of Abuja, Abuja, Nigeria

**Keywords:** Blood pressure-lowering, adherence clubs, Africa, primary care, medicines access

## Abstract

**Background:**

Hypertension control remains a significant challenge in reducing the cardiovascular disease burden worldwide. Community peer-support groups have been identified as a promising strategy to improve medication adherence and blood pressure (BP) control.

**Objectives:**

The study aimed to evaluate the feasibility and impact of adherence clubs to improve BP control in Southeast Nigeria.

**Methods:**

This was a mixed-methods research involving a formative (pre-implementation) research, pilot study and process evaluation. Hypertensive patients in two communities were recruited into peer-support adherence clubs under the leadership of role-model patients to motivate and facilitate medication adherence, BP monitoring, and monthly medication delivery for six months. The primary outcome was medication adherence measured using visual analogue scale (VAS), with BP level at six months as a key secondary outcome.

**Results:**

We recruited a total of 104 participants. The mean age was 56.8 (SD–10.7) years, 72 (69.2%) were women, mean BP was 146.7 (SD–20.1)/86.9 (SD–11.2) mmHg, and the mean percentage of medication adherence on the VAS was 41.4% (SD–11.9%). At six months, 67 patients were assessed; self-reported adherence on the VAS increased to 57.3% (SD–25.3%) (mean difference between baseline and follow-up of 15.5%, p < 0.0001), while the mean BP decreased to 132.3 (SD–22.0)/82.9 (SD–12.2) mmHg (mean difference of 13.0 mmHg in systolic BP, p < 0.0001 and of 3.6 mmHg in diastolic BP, p = 0.02). Five in-depth interviews and four focus groups discussions were conducted as part of the qualitative analyses of the study. The participants saw hypertension as a big issue, with many unaware of the diagnosis, and they accepted the CLUBMEDS differential service delivery (DSD) model concept in hypertension.

**Conclusions:**

The study demonstrates that the implementation of adherence clubs for hypertension control is feasible and led to a statistically significant and clinically meaningful improvement in self-reported medication adherence, resulting in BP reduction. Upscaling the intervention may be needed to confirm these findings.

## Introduction

Hypertension is the leading modifiable risk factor for cardiovascular diseases and a major public health issue globally, accounting for 10.8 million deaths in 2019 and resulting in a significant financial burden [1,2]. The global prevalence of hypertension was estimated to be about 1.3 billion adults in 2019 [3]. The problem is of particular importance in low- and middle-income countries (LMICs), where about 82% of hypertensive individuals live [3]. Despite the global effort to mitigate the impact of hypertension worldwide, blood pressure (BP) control remains unacceptably low, particularly in LMICs [2,4]. In terms of diagnosis, treatment and control, the global scenario is worrying. Globally, 59% of hypertensive women and 49% of hypertensive men are aware of their hypertension diagnosis; of those, only 47% of women and 38% of men are receiving treatment for hypertension [3]. Of those who are treated, only 23% of women and 18% of men have controlled BP, which is defined as BP levels lower than 140/90 mmHg [3,5].

Barriers to BP control exist at multiple levels, including health systems (e.g., long distance to health facilities, lack of anti-hypertensive drugs), communities, healthcare providers and patient related factors (e.g., lack of time due to competing priorities, health literacy) impeding hypertension control at the patient level [6,7]. Importantly, the World Health Organization (WHO), in HEARTS initiative, recommends the implementation of team-based care to engage patients and family members in the process of regularly checking BP and adhering to BP-lowering medications, with the aim to improve BP control [8,9,10]. In a 2013 report on hypertension, the WHO also emphasizes the importance of self-care and BP self-monitoring as a key strategy for the management of hypertension, especially where people have limited access to health services [11]. To address these barriers, community participation and patient ownership of care have been viewed as the key to better control of hypertension [6,12,13]. Therefore, innovations that empower the patients and the communities to take more responsibility in control and management of hypertension have the potential to go a long way in reversing the trend of the disease.

In this context, and aligned with the WHO global target of reducing the prevalence of hypertension by 25% by the year 2025 [14] and the World Heart Federation (WHF) target of improving hypertension awareness and control by 25% by the year 2025 [15], we designed the CLUBMEDS study to evaluate the feasibility and impact of community-based adherence clubs to improve hypertension control in Nigeria. The CLUBMEDS study was conceptualized by leveraging on the success of community-based strategies used in the management of HIV/AIDS, where patients were agents to improve disease awareness and medication adherence [16,17,18].

## Methods

The design and detailed methods of the CLUBMEDS study have been previously published [19]. In summary, the CLUBMEDS study was a mixed-methods research study that involved formative (pre-implementation) research, a pilot study and process evaluation.

### Formative research

In the formative research, we conducted a cross-sectional exploratory qualitative study among healthcare professionals involved in management of patients with hypertension at different levels with 5–30 years of experience. The topic guide included open-ended questions accompanied by follow-up probes. The main concept areas evaluated were barriers and facilitators, as well as opportunities and challenges for the implementation of the CLUBMEDS intervention, including the following topics:

1. Current understanding of hypertension
2. The challenges faced in accessing and adhering to medications by those who are using BP lowering medication
3. The perceived value and acceptability of CLUBMEDS strategy

All in-depth interview (IDI) sessions were audio-recorded and transcribed. We used a framework analysis method (a matrix-based approach) to identify existing and new patterns in the data [20]. This method was selected because it offers researchers a systematic structure to manage, analyse, and identify themes. Atlas.ti (version 8) software was used to assign open codes, including quotes (respondents’ exact words).

### The CLUBMEDS pilot study

#### Study population and intervention

The CLUBMEDS pilot study was designed to assess the impact of adherence clubs on hypertension control and medication adherence. At inception, our research team comprising of a physician, two research nurses, two field workers and a community health worker visited selected religious centers to raise awareness for the study and ask for permission to meet and recruit participants who had hypertension. Following the initial meeting, the research nurse obtained written informed consent from eligible participants, and they were assigned to respective clubs. The CLUBMEDS adherence clubs consisted of small groups of patients with hypertension led by a role-model patient, who acted as the club facilitator. Research nurses helped form the adherence clubs by selecting members based on their domiciliary proximity and one role-model patient for each club. To be eligible to be a club member, patients had to have a confirmed diagnosis of hypertension and have an indication for treatment with BP lowering medications, irrespective of their baseline adherence. In addition to that, to be a role-model patient, the participant had to have controlled BP <140/90mmHg, be adherent to their BP lowering treatment (defined by collection of the BP lowering medication every month in the last 6 months and attending the primary healthcare facility at least twice in the last 6 months), had to have received at least secondary education and agreed to take the responsibility of acting as the club facilitator. Primary healthcare facility nurses and community health workers (CHWs) were the point-of-contact for any clinical support needed by the club members. Before the initiation of the adherence clubs, the role-model patients and the CHWs received training on hypertension, including: the importance of regularly measuring BP and adhering to the treatment, how to measure BP with an automated BP monitor appropriately, and alert signs of uncontrolled symptomatic hypertension.

Once the adherence clubs were formed, the role-model patient and the club members were responsible for the overall running of the club meetings. These adherence club meetings were held once a month for 6 months in a local community center on a day and time decided by the members. These meetings were forums for the club members to have their BP checked, collect BP lowering medications, and discuss any issues related to medication adherence or worrying symptoms. Both participants with controlled and uncontrolled BP attended the group meetings for interaction and drug refill. However, those with persisting uncontrolled blood pressure, leg swelling, shortness of breath, limb weakness, or persisting cough were referred to the health facility for evaluation and returned to their clubs when stabilised.

The role-model patient was responsible for collecting the BP lowering medications at the facility and delivering them to the club members during the meetings. Participants still needed to pay for their medication, but the out-of-pocket cost was reduced. Role-model patients did not receive any payment for the facilitation of the club meetings. Club members returned to the primary healthcare facility if alert signs were present, for regular medical follow-up, medication prescription and up-titration or change in their BP lowering medication regimen, as needed or after six months if they remained stable.

#### Data collection and study outcomes

The primary outcome of the study was medication adherence evaluated using a visual analogue scale (VAS) at six months. Secondary outcome measures were also measured at six months. The key secondary outcome was BP (measured by the research nurse), and other secondary outcomes included medication adherence measured by the number of pills missed in the last seven days (self-report) and pill count (by research nurse or role-model patient), self-reported hospital admissions due to cardiovascular events, attendance at CLUBMEDS meetings and acceptability of the CLUBMEDS strategy by relevant stakeholders.

Potential participants were evaluated for participation in the study by a research nurse who collected the study data. At baseline, data on sociodemographic characteristics including age, sex, marital status, education, employment status, income and main occupation were collected, as well as data on prior medical history, smoking and alcohol consumption, height and weight. Data on the BP lowering medications in use and medication adherence were collected at both baseline and 6-month follow-up. Also, BP and heart rate measurements were performed at both time points. Three BP measurements were performed using an automated BP monitor (Omron model 705IT) using appropriate cuff size, after five minutes rest with the hands on the table, feet on the floor and back rested on the chair. We chose automatic BP measuring over manual in line with evidence that values from the former were more reproducible [21,22,23]. After the six adherence club meetings, an end-of-study follow-up visit was scheduled at the primary healthcare facility for outcome assessment and for returning to usual care. Poor attendance at the final visit led to investigators offering home visits when needed to complete the end-of-study procedures.

#### Analyses

Statistical analyses were conducted using SAS Enterprise Guide 7.1 (SAS Institute Inc., Cary, NC, USA). Baseline data were presented for continuous variables as means and standard deviations, while for categorical variables, frequencies and percentages were used to represent the data. BP was analyzed, excluding the first BP measurement, and calculating the mean of the second and third measurements. A change >5mmHg is considered clinically significant [24,25].

We analyzed the study outcomes using a pre-post analysis method. The student t-test (continuous variables) and χ^2^ tests (categorical variables) were used to compare the study outcomes between baseline and 6-months. For non-normally distributed data, we used Mann-Whitney tests.

We used VAS as a continuous variable in a linear regression model to study essential determinants of medication adherence. However, there was insufficient data to carry out the planned analysis using the pre-defined 80% threshold on the VAS.

### Process evaluation

As part of the process evaluation, focus group discussions (FGDs) were conducted using a topic guide—which included open-ended questions accompanied by follow-up probes—to refine the feasibility of study design and collect feedback and participant experiences about the CLUBMEDS model. These FGDs were conducted between baseline and follow-up assessment at 6 months.

All FGDs were audio-recorded and transcribed. We also used a framework analysis method to identify existing and new patterns in the data. Atlas.ti (version 8) software was also used to assign open codes in this evaluation.

### Ethical Considerations

This study was conducted following the Declaration of Helsinki, a statement of ethical principles for medical research involving human subjects. The study was approved by the University of Abuja Teaching Hospital (Ref. no. UATH/HREC/PR/2017/012/001) and the Federal Teaching Hospital Abakaliki Human Research Ethics Committees (Ref. no. FETHA/REC/VOL2/2018/008). All participants provided written informed consent to study research nurse and their data were de-identified and entered in a secure password-protected database. The managing physicians were informed of their patients’ participation in the study, and each participant was free to decline participation with no consequence on their continued care.

## Results

### Clubmeds pilot study results

Participant enrolment was performed between March and September 2018 in two primary healthcare facilities, one urban (Abakaliki, the capital of Ebonyi state) and one rural (Awkuzu, a town in Oyi Local Government Area of Anambra State), in Southeast Nigeria. A total of 104 participants were recruited from the two sites, and the last follow-up assessment was performed in May 2019. Nine adherence clubs were formed, seven in the urban site and two in the rural site, with between 10 to 13 club members in each club. However, two of the urban site clubs had to be merged into one club due to poor attendance. Meetings were scheduled to coincide with monthly religious meetings as this was convenient for the participants. At baseline, the participants’ mean age was 56.8 years (standard deviation [SD] 10.7), ranging from 33 to 85 years, and 69.2% were women. The mean percentage of medications taken measured by the VAS was 41.4% (SD 11.9), while BP was 146.7 (SD 20.1)/86.9 (SD 11.2) at baseline. ***Table 1*** shows the baseline characteristics overall and per study site. At six months, 67 patients (64% of the initial cohort) were assessed: 25 participants during the facility follow up visits and 42 during home visits (of which two did not complete outcome measurements).

**Table 1:** Baseline characteristics of participants in the medication adherence clubs.


CHARACTERISTICS	RURAL SITE (N = 22)	URBAN SITE (N = 82)	OVERALL (N = 104)

**Demographics**			

Age, years – mean (SD)	65.6 (9.3)	54.4 (9.8)	56.8 (10.7)

Female – n (%)	15 (68.2)	57 (69.5)	72 (69.2)

Marital status – n (%)			

Married	18 (81.8)	72 (87.8)	90 (86.5)

Widowed	4 (18.2)	10 (12.2)	14 (13.5)

Level of Education – n (%)			

None	2 (9.1)	2 (2.4)	4 (3.9)

Primary school	9 (40.9)	29 (35.4)	38 (36.5)

Secondary school	9 (40.9)	35 (42.7)	44 (42.3)

Post-secondary or tertiary education	2 (9.1)	16 (19.5)	18 (17.3)

Employment Status – n (%)			

Currently employed	4 (18.2)	40 (48.8)	44 (42.3)

Unemployed	13 (59.1)	19 (23.2)	32 (30.8)

Retired	1 (4.5)	4 (4.8)	5 (4.8)

Never worked	4 (18.2)	19 (23.2)	23 (22.1)

**Comorbidities and other risk factors**			

Prior myocardial infarction– n (%)	0	0	0

Prior stroke– n (%)	0	0	0

Prior diagnosis of peripheral artery disease– n (%)	0	1 (1.2)	1 (0.9)

Prior diagnosis of heart failure– n (%)	0	0	0

Diabetes – n (%)	1 (4.5)	9 (11.0)	10 (9.6)

Chronic kidney disease – n (%)	0	1 (1.2)	1 (0.9)

Smoking – n (%)			

Current smoker	0	0	0

Ex-smoker	1 (4.5)	1 (1.2)	2 (1.9)

**Clinical data**			

BMI, kg/m^2^ – mean (SD)	29.4 (9.2)	27.8 (5.1)	28.2 (6.2)

Systolic BP, mmHg – mean (SD)	141.5 (18.4)	148.1 (20.4)	146.7 (20.1)

Diastolic BP, mmHg – mean (SD)	86.0 (9.8)	87.2 (11.5)	86.9 (11.2)

Heart rate, bpm – mean (SD)	73.1 (8.7)	80.0 (12.0)	78.5 (11.6)

**BP lowering therapy**			

Number of classes – mean (SD)	2.14 (0.56)	1.67 (0.63)	1.77 (0.64)

1 drug prescribed – n (%)	2 (9.1%)	33 (40.2%)	35 (33.7%)

2 drugs prescribed – n (%)	15 (68.2%)	44 (53.7%)	59 (56.7%)

≥ 3 drugs prescribed – n (%)	5 (22.7%)	5 (6.1%)	10 (9.6%)

Blood pressure lowering classes			

Thiazide diuretic	5 (22.7%)	46 (56.1%)	51 (49.0%)

Thiazide diuretic + potassium sparing diuretic	11 (50.0%)	9 (11.0%)	20 (19.2%)

ACE inhibitor or ARB	0	25 (30.5%)	25 (24.0%)

Calcium channel blockers	14 (63.6%)	50 (61.0%)	64 (61.5%)

**Medication adherence**			

Visual Analogue scale, % of medication taken – mean (SD)	42.4 (12.8)	41.4 (11.6)	41.4 (11.9)


ACE, Angiotensin conversing enzyme; ARB, Angiotensin II receptor blocker; BMI, body mass index; BP, blood pressure; SD, standard deviation.

#### Visual analogue scale and blood pressure

Among the 25 patients who attended the facility follow-up visits, compared to baseline, at six months, there was a significant improvement in medication adherence as measured by the VAS with an increase of 25.0% (SD 22.1) in the percentage of medications taken in the past month (p < 0.0001) (***Table 2***). There was also a significant decrease of 9.4mmHg (SD 17.9, p = 0.0149) in systolic BP and a non-significant decrease of 1.6 mmHg (SD 10.3, p = 0.4353) in diastolic BP between baseline and 6-months follow up assessment.

**Table 2:** Changes in medication adherence and blood pressure over time.


	BASELINE	6-MONTHS FOLLOW-UP ASSESSMENT	MEAN DIFFERENCE	P VALUE

**Facility follow-up visits (n = 25)**				

**Primary outcome**				

Visual Analogue scale, % of medication taken – mean (SD)	41.4 (11.9)	66.54 (21.0)	25.0 (22.1)	<0.0001

**Key secondary outcome**				

Systolic BP, mmHg – mean (SD)	146.7 (20.1)	130.96 (16.9)	9.4 (17.9)	0.0149

Diastolic BP, mmHg – mean (SD)	86.9 (11.2)	81.9 (10.0)	1.6 (10.3)	0.4353

**All follow-ups (facility follow-up visits + home visits) (n = 65)**				

**Primary outcome**				

Visual Analogue scale, % of medication taken – mean (SD)	41.4 (11.9)	57.3 (25.3)	15.5 (28.3)	<0.0001

**Key secondary outcome**				

Systolic BP, mmHg – mean (SD)	146.7 (20.1)	132.3 (22.0)	13.0 (20.9)	<0.0001

Diastolic BP, mmHg – mean (SD)	86.9 (11.2)	82.9 (12.2)	3. 6 (12.1)	0.0202


Pooling the facility and home end-of-study measurements allowed 65 participants to be included in the outcome assessment. There was a significant increase of 15.5% (SD 28.3) in the proportion of medication taken in the past month (p < 0.0001) as reported in the VAS. In addition, these results showed a more substantial decrease in systolic BP of 13.0 mmHg (SD 20.9, p < 0.0001) and a significant decrease of 3.6 mmHg (SD 12.1, p = 0.0202) in diastolic BP. Forty-six (71%) participants who completed either facility or home follow-up had controlled blood pressure <140/90mmHg; of these, 28 were not controlled at baseline. Thirteen participants did not have controlled blood pressure at either baseline or at 6-months.

#### Medication adherence by number of pills missed and pill count

Medication adherence was also assessed by asking the patient the number of anti-hypertensive pills missed in the last 7 days and by pill count. At baseline, the mean number of pills missed in the last 7 days was 3.3 pills (SD 1.59). At six months, considering only the facility follow-up visits, this number significantly decreased to 1.19 (SD 2.08), with a mean difference of 1.73 pills (SD 2.44, p = 0.0013). Meanwhile, considering all follow-up visits (both facility based and home visits), the mean number of pills missed in the last 7 days was 2.17 pills (SD 2.78), with a mean difference of 1.04 pills (SD 2.99, p = 0.0061). Based on pill counts, mean adherence was 97% (SD 10%) after the first meeting (n = 61) and 100% (SD 0%) after the fifth meeting (n = 23) (Supplementary table 1).

#### Hospital admissions

During the follow-ups, only one patient from the urban site reported being hospitalized due to heart failure, and another patient from the rural site due to a non-cardiovascular cause.

#### Attendance

**Figure 1:**
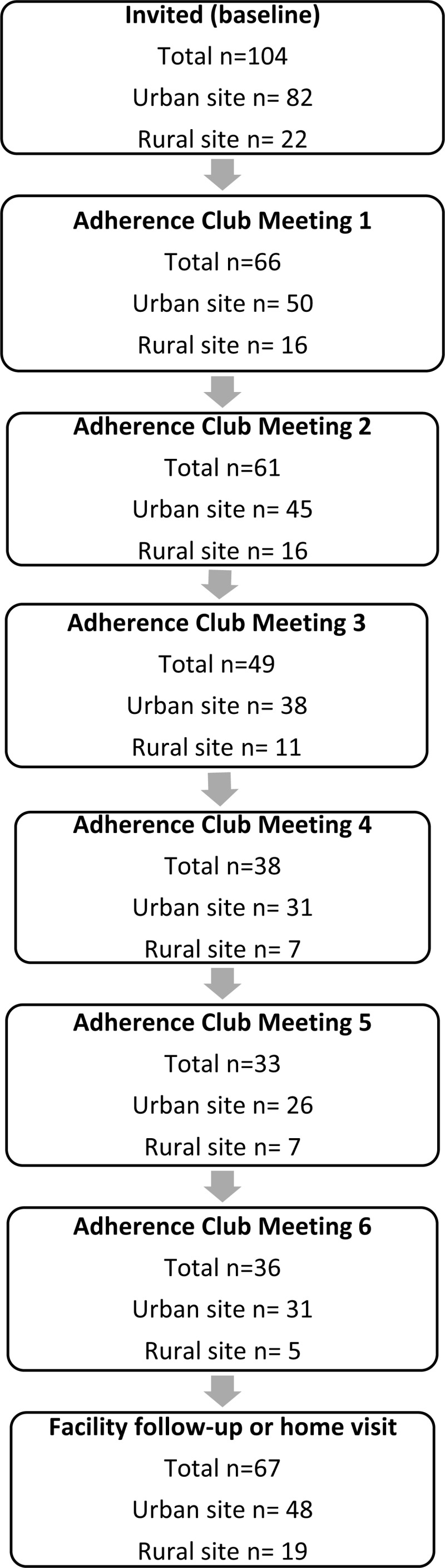
Flowchart of number of patients invited, attending each adherence club meeting, and attending facility follow-up or home visit.

Attendance to the adherence clubs’ meetings during the 6 months of intervention was varied, with a trend to decreasing numbers over time (***Figure 1***). Thirty patients invited to participate never attended any adherence club meetings, 25 in the urban site and 5 in the rural site. As mentioned, at the end of 6 months, only 25 participants returned to a facility follow-up visit and another 45 participants were assessed during home visits. The main reason given for missing the follow-up visit was family or personal commitments followed by work commitments, but it is notable that long travel distances was another common reason in the rural site (Supplementary table 2). Exploratory analyses were performed, comparing the baseline characteristics of those who completed the follow-up assessment with those who did not to understand if there were any relevant characteristic related to follow-up completion. We found that, compared to those who completed the follow-up assessment, those who did not complete were older (59 vs. 56 years), mostly men (41% vs. 25%), had lower education levels (59% vs. 49% with secondary school certificate or less) and had higher BP levels at baseline (149/88 mmHg vs. 145/86 mmHg); however, these differences were not statistically significant.

### Qualitative assessment

#### Formative research

The formative research was conducted between July and August 2018. Five IDIs were conducted with relevant stakeholders (different categories of health workers made up of doctors, nurses, community pharmacists, administrators) in the same study sites as the pilot study (Supplementary table 3). Each interview lasted approximately one hour.

The qualitative data analyses revealed three main themes related to participant’s views and concerns related to hypertension and the feasibility of the CLUBMEDS model. These included current understanding, barriers for medication adherence and treatment and perception about CLUBMEDS model.

Regarding the current understanding about the disease, the study participants described hypertension as a big issue affecting their community and that the situation was worse because many people were not aware that they were affected by hypertension. The participants suggested that the government should give equal attention to non-communicable (NCDs) and communicable diseases, since NCDs have reached to an epidemic proportion in Nigeria.

*“There are so many patients with hypertension, and it is difficult to put a figure to it” (Pharmacist, Urban site IDI)*

Financial constraints and poor health-seeking behavior appeared to be major barriers to accessing regular medication to treat hypertension. Four study participants, including the NCD director, the primary health care director, a nurse, and the pharmacist, mentioned that drug cost is a major obstacle for regular medicine adherence by the patients. A nurse from the urban site mentioned that in addition to financial constraints, poor health-seeking behavior is the reason for the non-adherence to anti-hypertensive medications.

*“May be some of them don’t adhere to their medications due to ignorance, not knowing that stopping drugs could result in complication; transportation can also be an issue, also cost of the drugs. Some if you send them to collect the drug, they will tell you they don’t have the money to collect the drugs. Many believe that taking drug once can cure them and they do not continue treatment” (Nurse, Urban site IDI)*

Concerning perception about the CLUBMEDS model, a nurse working in the rural area mentioned that CLUBMEDS could potentially be very effective in terms of raising awareness and improvement of hypertension management. Another nurse working in the urban area also expressed that CLUBMEDS will be helpful to control hypertension in the area like a support group system helped to control HIV in the community. A pharmacist working in the urban area said that the CLUBMEDS model is good for accessibility of drugs, but it cannot replace the doctors. The study participants suggested strict monitoring and an evaluation framework to improve the model.

*“There is need for strict monitoring and evaluation framework in ensuring control example having a professional looking at each location” (NCD Director, Rural site, IDI)*

*“If you people can have people in the village identifying the people with BP, then like in HIV the problem of BP can be stopped” (NCD Director, Rural site, IDI)*

#### Process evaluation

For the process evaluation, 4 FGDs were conducted (***Table 3***), two each, at the urban site (total of 39 participants) and the rural site (total of 25 participants). The first discussions occurred in July/August 2018 and the second discussions occurred in January/February 2019.

**Table 3:** Focus group discussion participants characteristics.


STUDY SITE LOCATION	STUDY PARTICIPANTS AT FIRST DISCUSSION	INTERVIEW METHOD	STUDY PARTICIPANTS AT SECOND DISCUSSION	INTERVIEW METHOD

**Urban site**	17 participants (8 women and 9 men)	FGD1	22 participants (12 women and 10 men)	FGD3

**Rural site**	21 participants (11 women and 10 men)	FGD2	4 participants (3 women and 1 man)	FGD4

**Total**	**38 (19 women and 19 men)**		**26 (15 women and 11 men)**	


FDG, focus group discussion.

The study participants shared their experience about CLUBMEDS model as it was being implemented; some patients mentioned that CLUBMEDS has helped them to manage hypertension more effectively. One patient said he was afraid, because he did not have personal contact with any doctor, but now he is happy to see a nurse, who supported the CLUBMEDS model. According to a patient, the drugs can be accessible at a low cost from the CLUBMEDS.

*“It is very helpful to us and we are very happy”* (*Patient, Urban site, FGD1)*

*“Yes, it is a good one”* (*Patient, Rural site, FGD2)*

*“When I heard of CLUBMEDS, I decided to join and have been counselled on regular check and drug use” (Patient, Urban site, FGD1)*

*“I have also learnt a lot on how to take my drugs from CLUBMEDS. What I understood now is the need to spread my drugs for better control” (Patient, Urban site, FGD1)*

*“In my suggestion, it is to consider if the drugs can be delivered through the role model patient since the primary health care is increasing the cost” (Patient, Rural site, FGD2)*

## Discussion

Improving access to essential cardiovascular medicines is vital to achieving the WHF’s 25 × 25 targets for reducing premature mortality from cardiovascular disease. Several policies and strategies have been employed in improving access to essential medicines in LMICs with limited success [26]. In the CLUBMEDS study, adherence clubs were designed to improve access and adherence to BP lowering medications, as well as BP control, learning from similar strategies employed in HIV/AIDS context [19].

In this feasibility study, we found that, at the 6-months follow up assessment, there was a clinically meaningful and statistically significant improvement in self-reported medication adherence with a consequent reduction in BP. Similar findings were obtained using a more objective assessment of medication adherence using pill counts during the club’s meetings in the study. However, the attendance to the clubs’ meetings diminished with the duration of the study.

Findings from qualitative research highlight the participants’ understanding and acceptability of the CLUBMEDS model. This was considered similar to the support group system [27] of patients with HIV/AIDS to provide links between the patients and the health care system. The qualitative data analysis provides a closer look at the challenges for patients in accessing care and adhering to the treatment. As in other studies [28,29,30,31], the present study found that drug cost, limited understanding and low health-seeking behavior are the main reasons for non-adherence to BP lowering medications.

We observed that empowering patients to take a larger role in the management of hypertension is feasible and improved self-reported adherence. For this to be successful it was crucial for patients to gain ownership of their BP, with peer support and leadership from role model patients, and guidance of nurses and community health workers. Differentiated service delivery has been shown to improve care in HIV/AIDS setting [32], and promising evidence from NCD context has shown that similar interventions are equally effective for the management of chronic diseases. One study from rural South Africa with 224 patients found that task shifting for hypertension, diabetes and asthma, an intervention driven by nurses with physician support, led to improved adherence to medications and better control [33]. Likewise, other interventions in low-income settings showed that lay health workers can be an important resource in hypertension and diabetes management [34,35]. Expanding the findings from these studies, our study reinforces the notion that models that engage the community and increase access to BP lowering medications in the community by reducing visits to the clinics to pick up medications can achieve improved adherence and better control of hypertension.

It is important to mention that the study findings should be interpreted in the context of the study’s pre-post design, which precludes comparison to a control group. The changes observed could be due to factors other than participation in the adherence clubs, including regression to the mean [36,37]. Furthermore, those who remained in the study may be more adherent to blood pressure lowering medications than those lost to follow-up. We observed diminished attendance to the club meetings over time, which may be due to reasons such as: i) improvement in BP control with a subsequent attenuation of perceived need to attend the club meetings, ii)non-specific symptoms or asymptomatic nature of hypertension [38]. In addition, some sociodemographic characteristics, such as older age, male sex and lower education, might be associated with poorer engagement with the intervention. To overcome this diminished attendance, we made multiple attempts to collect data for those who did not attend the final club meeting, including home visits.

The study has strengths and limitations. A strength of this study was the early engagement with individuals with hypertension, health care professionals, health service providers and decision-makers, which led to greater community ownership and acceptability of the intervention by study participants and stakeholders. Limitations of this study include not having a comparator group, the small sample size, short follow-up period and the study attrition. Though not evaluated in our study, initial blood pressure control (yes, no) may impact future blood pressure outcomes. Therefore, future research is needed to explore how patients move across care pathways, including any differential impact of initial control status (yes, no) onto blood pressure outcomes, including adherence and control during follow up. A larger cohort of participants over a longer period compared to standard care may be required to further demonstrate retention and effectiveness of the medication adherence clubs for improving blood pressure control, and impact on health service load in LMICs.

## Conclusion

The CLUBMEDS study demonstrated the feasibility and acceptability of medication adherence clubs for BP management in Southeast Nigeria. During the study period of 6 months, BP reduced from 147/87mmHg to 131/82mmHg and self-reported adherence using the VAS increased from 41% to 57%. Upscaling the research over a longer period may further establish effectiveness of the intervention and determine qualitative impact on health service load and patient experiences in several different settings.

## Additional File

The additional file for this article can be found as follows:

10.5334/gh.1109.s1 Supplementary appendix. Supplementary Tables 1–4.
