# Supplementary material for: Adherence Clubs to Improve Hypertension Management in Nigeria: Clubmeds, a Feasibility Study
Source: Glob Heart. 2022 Mar 16;17(1):21. doi: 10.5334/gh.1109 (PMC8932363; doi:10.5334/gh.1109)
Supplement: Supplementary appendix. — Supplementary Tables 1–4. [file gh-17-1-1109-s1.pdf]

Supplementary appendix

Supplementary Table 1: Medication adherence by blood pressure-lowering pill count

Adherence club meeting	Total number of attendees	Mean adherence (SD)	Minimum	Maximum
After 1st meeting	61	97% (10%)	33%	100%
After 2nd meeting	49	98% (7%)	60%	100%
After 3rd meeting	38	99% (8%)	50%	100%
After 4th meeting	29	100% (2%)	87%	100%
After 5th meeting	23	100% (0%)	100%	100%

Supplementary Table 2: Reason given for missing follow-up visit during home visit

	Rural	Urban
Personal/family commitments	3	16
Work commitments	4	9
Long travel distances	4	0
Time constraints	1	0
Other	2	3
**Total**	**14**	**28**

Supplementary Table 3: Formative research study participants details

Study site location	Study participants	Interview method
**Urban site**	Primary Health Care Director Ebonyi state	in-depth interview
	Nurse	in-depth interview
	Pharmacist	in-depth interview
**Rural site**	Nurse	in-depth interview
	NCD Director Anambra state	in-depth interview
**Total**	**5 (2 Women and 3 Men)**	**5 interviews**

Supplementary Table 4: Blood pressure at beginning and end of study in relation to target

	End blood pressure		Total
	At target	Not at target	
Baseline blood pressure	18	6	24
At target			
Not at target	28	13	41
Total	46	19	65

Notes: 39 missing end-of-study blood pressure measures not shown in this table. “At target” is defined as SBP<140 and DBP<90 mmHg, and “Not at target” is defined as SBP ≥140 or DBP≥90 mmHg. DBP= diastolic blood pressure; SBP= systolic blood pressure.
